# Genetic Variants in TGF-β Pathway Are Associated with Ovarian Cancer Risk

**DOI:** 10.1371/journal.pone.0025559

**Published:** 2011-09-30

**Authors:** Jikai Yin, Karen Lu, Jie Lin, Lei Wu, Michelle A. T. Hildebrandt, David W. Chang, Larissa Meyer, Xifeng Wu, Dong Liang

**Affiliations:** 1 Department of Epidemiology, The University of Texas MD Anderson Cancer Center, Houston, Texas, United States of America; 2 Department of Gynecologic Oncology, The University of Texas MD Anderson Cancer Center, Houston, Texas, United States of America; 3 Department of Pharmacology and Pharmaceutical Sciences, University of Houston, Houston, Texas, United States of America; 4 College of Pharmacy, Texas Southern University, Houston, Texas, United States of America; Institut Jacques Monod, France

## Abstract

The transforming growth factor-β (TGF-β) signaling pathway is involved in a diverse array of cellular processes responsible for tumorigenesis. In this case-control study, we applied a pathway-based approach to evaluate single-nucleotide polymorphisms (SNPs) in the TGF-β signaling pathway as predictors of ovarian cancer risk. We systematically genotyped 218 SNPs from 21 genes in the TGF-β signaling pathway in 417 ovarian cancer cases and 417 matched control subjects. We analyzed the associations of these SNPs with ovarian cancer risk, performed haplotype analysis and identified potential cumulative effects of genetic variants. We also performed analysis to identify higher-order gene-gene interactions influencing ovarian cancer risk. Individual SNP analysis showed that the most significant SNP was *SMAD6*: rs4147407, with an adjusted odds ratio (OR) of 1.60 (95% confidence interval [CI], 1.14–2.24, *P* = 0.0066). Cumulative genotype analysis of 13 SNPs with significant main effects exhibited a clear dose-response trend of escalating risk with increasing number of unfavorable genotypes. In gene-based analysis, *SMAD6* was identified as the most significant gene associated with ovarian cancer risk. Haplotype analysis further revealed that two haplotype blocks within *SMAD6* were significantly associated with decreased ovarian cancer risk, as compared to the most common haplotype. Gene-gene interaction analysis further categorized the study population into subgroups with different ovarian cancer risk. Our findings suggest that genetic variants in the TGF-β signaling pathway are associated with ovarian cancer risk and may facilitate the identification of high-risk subgroups in the general population.

## Introduction

Ovarian cancer is the leading cause of death from gynecologic cancer among women in the United States, with an estimated 21,880 new cases and 13,850 deaths in 2010 [1]. Because the disease is mostly symptomless in early stages and there are currently no effective screening methods, 75% of women present with advanced-stage disease (stage III or IV). The 5-year survival rate of advanced-stage disease is only around 30% [2]. The etiology of ovarian cancer remains largely unknown, although hormonal factors, inflammation, and wound healing are thought to play important roles [3].

Ovarian cancer is a multifactorial disease and genetic susceptibility has been suggested in previous studies. For example, mutations in *BRCA1*, *BRCA2*, *MLH1*, and *MSH2* were found to account for approximately 50% of familial ovarian cancers [4], [5]. However, there are compelling evidence suggesting that common genetic variants contribute to ovarian cancer susceptibility [6], [7]. Recently, genome-wide association studies (GWAs) have identified several common susceptibility alleles in four loci showing strong associations, but as most SNPs identified in GWAs, the associations are usually low in magnitude with most of the ORs less than 1.3 [8], [9], [10]. Due to the heterogeneous and multigenic nature of ovarian cancer, it is unlikely that any single SNP will be sufficient to confer disease risk. A comprehensive pathway-based analysis that focuses on evaluating the cumulative effects of a panel of SNPs would be more powerful to pinpoint the susceptibility genes and polymorphisms.

The transforming growth factor-β (TGF-β) pathway, including TGF-βs, bone morphogenetic proteins (BMPs), activins, and related proteins, is involved in a diverse array of cellular processes, including cell proliferation, morphogenesis, migration, extracellular matrix production, and apoptosis. Alteration of TGF-β superfamily signaling has been implicated in various human pathologies, including cancer, developmental disorders, cardiovascular and autoimmune diseases [11], [12], [13]. Experimental data have shown that more than 75% of human ovarian cancers exhibit resistance to TGF-β signaling [14], [15], suggesting that diminished TGF-β responsiveness is a key event in this disease. In normal ovarian surface epithelial cells, autocrine growth inhibition is maintained by TGF-β [16], but tumor cells escape the antiproliferative effects of TGF-β by acquiring mutations in the components of the signaling pathways or by selectively disrupting TGF-β. Mutations and deletions of Smad genes in the TGF-β signaling pathway often lead to unstable protein products that are rapidly degraded after ubiquitination and shift the equilibrium of the signaling cascade resulting in tumorigenesis [11]. Studies have reported the presence of some common genetic variations in the TGF-β signaling pathway to be related to ovarian carcinogenesis, such as *TGFB1*: rs56361919 in 23% of ovarian cancer cases [17]. In addition, mutations and/or alterations in the expression of TGF-β receptors and loss of SMAD4 are frequently detected in human ovarian tumors [18].

Given the critical role of the TGF-β pathway in maintaining proper cellular function and the disruption of this pathway in ovarian cancer, it is possible that common genetic variations in this pathway may affect the risk of ovarian cancer. To our knowledge, no molecular epidemiologic studies have been performed to comprehensively evaluate genetic variants in this pathway with ovarian cancer risk. In this study, we aimed to test the hypothesis that common germline genetic variants in the TGF-β pathway are associated with ovarian cancer risk.

## Methods

### Study population and data collection

The patient population has been described previously [19]. Briefly, 417 newly diagnosed and histologically confirmed ovarian cancer patients with primary malignancy were recruited at The University of Texas MD Anderson Cancer Center. Cases had not received any chemotherapy or radiotherapy prior to recruitment. There were no restrictions on recruitment in terms of age, ethnicity, or clinical stage of disease. The 417 controls were healthy women without prior history of cancer (except nonmelanoma skin cancer) and identified from a large pool of control subjects enrolled in on-going case-control studies of cancer. Controls subjects were individuals seeing a physician for routine health checkups or addressing health concerns at the Kelsey–Seybold Clinic. Cases and controls were matched by age (±5 years) and ethnicity.

Demographic characteristics (age and ethnicity), occupational history, tobacco use history, medical history, family history of cancer, and other epidemiologic data were collected for all patients and controls. For each participant, a blood sample was collected into heparinized tubes for lymphocyte isolation and DNA extraction. For all cases and controls, a written informed consent was obtained prior to participation and the donation of blood samples. The study was approved by Institutional Review Boards of MD Anderson and Kelsey Seybold Clinic.

### SNP Selection and Genotyping

The procedures used to select SNPs of the TGF-β pathway have been described previously [20]. Briefly, we compiled data from Gene Ontology (http://www.geneontology.org) and a systematic literature review to refine the gene list in the TGF-β signaling pathway. Tagging SNPs were identified from the HapMap database (http://www.hapmap.org) and selected using LDSelect program (http://droog.gs.washington.edu/ldSelect.html) to separate SNPs into bins on the basis of linkage disequilibrium. Selected tagging SNPs have a r^2^ threshold of 0.8, minor allele frequency (MAF) greater than 0.01 in Caucasian population and are located within 10 kb upstream of transcriptional start site and 10 kb downstream of transcriptional end site. Potentially functional SNPs (e.g., coding SNPs and SNPs in untranslated regions, promoters, and splicing sites) were also included. Overall, 218 SNPs in 21 genes of the TGF- β pathway were selected along with SNPs from other cancer-related pathways. Complete set of SNPs was sent to Illumina technical support for custom iSelect, Infinium II BeadChip design using proprietary program developed by Illumina. Genomic DNA was isolated from peripheral blood lymphocytes using the QIAamp DNA Blood Maxi kit (QIAGEN, Valencia, CA). Genotyping followed the standard protocol of Illumina's Infinium iSelect HD Custom Genotyping Beadchip provided by Illumina (San Diego, CA). BeadStudio software was used to call genotypes. All laboratory personnel were blinded to the case-control status of the study subjects.

### Statistical Analyses

The distribution of categorical variables and continuous variables between cases and control subjects was compared by Pearson's χ^2^ test and Student's *t* test, respectively. For each SNP in this study, we tested Hardy-Weinberg equilibrium using the goodness-of-fit χ^2^ test to compare the observed with the expected frequency of genotypes in control subjects. For SNP analysis, we tested three different genetic models, dominant model, recessive model and additive model to identify the best-fitting model with the smallest *P* value. If the percentage of the homozygous variant genotypes was less than five in cases or controls, we only considered the dominant model which has the highest statistical power. Multiple logistic regression analysis was used to estimate the odds ratios (ORs) and 95% confidence intervals (CI) while adjusting for age and ethnicity where appropriate. For internal validation, a bootstrap resampling method was performed 100 times on samples randomly drawn from the original data set and a *P* values was obtained for the best-fitting model in each bootstrapped sample. Cumulative effects of SNPs were assessed by summing up the putative unfavorable genotypes showing significant association with the risk (P<0.05) in single SNP analysis and then grouped into four categories based on the distribution of ORs. A gene-based analysis was used to explore the associations between genes and ovarian cancer risk using the likelihood-ratio test (LRT) as described previously [21]. Classification and regression tree (CART) analysis was used to explore higher-order gene-gene interactions using the Expectation-Haplotype analysis was performed using the maximization algorithm implemented in the HelixTree software (Golden Helix, Bozeman, MT). We also performed 10,000 bootstrap runs to construct 95%CIs for the ORs in cumulative genotype analysis and CART analysis. All statistical analyses were adjusted for age, ethnicity. Statistical analysis was performed using STATA 10.0 (College Station, TX).

## Results

### Subject characteristics

In this study, there were 417 cases and 417 age- and ethnicity-matched control subjects. The mean age was 60.73 (SD: 10.36) in cases and 60.30 (SD: 10.71) in control subjects (*P* = 0.554). The majority of the cases (n = 339, 81.29%) and controls (n = 349, 83.69%) were Caucasians. Of the cases, the majority are diagnosed at stage III (66.5%), whose tumors are of the serous subtype (61.3%) (**Table S1**).

### Association between individual SNP and risk

A total of 218 SNPs from 21 genes in the TGF-β pathway were analyzed (**Table S2**). Twenty-three SNPs from ten genes showed significant associations with ovarian cancer risk at *P*<0.05 (**Table 1**). Internal validation by bootstrapping method identified 13 SNPs from eight genes showing consistent associations (i.e. *P*<0.05 in 80 or more among 100 bootstrapped samples). The most significant SNP was *SMAD6*: rs4147407 with subjects carrying at least one variant allele exhibiting a 1.60-fold increased risk (95%CI, 1.14–2.24). For another SNP in *SMAD6*, the variant allele of rs4075546 was associated with decreased risk (OR, 0.77; 95%CI, 0.63–0.94, *P* = 0.0099).

**Table 1:** Associations between TGF-β Pathway SNPs and ovarian cancer risk.

Gene	SNP	Genotype (case/control)				OR (95% CI)*	Model**	*P*	Bootstrap*P*<.05
		WW (n/n)	WV (n/n)	VV (n/n)	MAF				
***BMP2***	**rs235757**	**174/157**	**198/192**	**45/68**	**0.35/0.39**	**0.64 (0.42–0.95)**	**REC**	**0.029**	**96**
*INHA*	rs7588807	107/82	216/241	94/94	0.48/0.51	0.70 (0.50–0.98)	DOM	0.035	64
***INHBC***	**rs2228225**	**131/171**	**203/174**	**83/72**	**0.44/0.38**	**1.48 (1.11–1.99)**	**DOM**	**0.008**	**100**
	**rs4760259**	**165/199**	**199/171**	**53/47**	**0.37/0.32**	**1.39 (1.06–1.84)**	**DOM**	**0.019**	**95**
***SMAD1***	**rs11724777**	**160/155**	**200/177**	**57/85**	**0.38/0.42**	**0.63 (0.43–0.91)**	**REC**	**0.014**	**100**
	rs6537355	340/319	69/93	8/4	0.10/0.12	0.69 (0.49–0.98)	DOM	0.036	11
***SMAD2***	**rs1792689**	**328/301**	**87/105**	**2/11**	**0.11/0.15**	**0.69 (0.50–0.95)**	**DOM**	**0.024**	**85**
	rs1792658	256/228	138/166	19/19	0.21/0.25	0.75 (0.56–0.99)	DOM	0.043	55
***SMAD3***	**rs10152307**	**243/206**	**140/179**	**34/32**	**0.25/0.29**	**0.72 (0.54–0.95)**	**DOM**	**0.019**	**100**
	rs4776892	269/244	123/155	25/18	0.21/0.23	0.73 (0.55–0.98)	DOM	0.035	28
	**rs7183244**	**192/160**	**167/186**	**58/71**	**0.34/0.39**	**0.74 (0.56–0.98)**	**DOM**	**0.035**	**88**
***SMAD6***	**rs4147407**	**313/343**	**100/69**	**4/5**	**0.13/0.09**	**1.60 (1.14–2.24)**	**DOM**	**0.007**	**100**
	**rs4075546**	**185/152**	**185/199**	**47/66**	**0.33/0.40**	**0.77 (0.63–0.94)**	**ADD**	**9.9×10^−3^**	**100**
	**rs16953584**	**258/235**	**144/150**	**15/31**	**0.21/0.25**	**0.45 (0.24–0.87)**	**REC**	**0.016**	**95**
	rs2053424	139/166	212/182	66/68	0.41/0.38	1.43 (1.07–1.92)	DOM	0.017	51
	rs5014202	278/251	125/145	14/21	0.18/0.22	0.78 (0.61**–**0.99)	ADD	0.040	67
	**rs4776318**	**140/116**	**198/201**	**79/100**	**0.43/0.48**	**0.82 (0.68–1.00)**	**ADD**	**0.047**	**83**
***SMAD7***	**rs17186485**	**370/346**	**47/67**	**0/4**	**0.06/0.09**	**0.63 (0.42–0.93)**	**DOM**	**0.021**	**94**
	rs3736242	229/259	168/137	18/21	0.25/0.21	1.37 (1.03–1.81)	DOM	0.029	51
	rs7238442	124/120	206/193	84/104	0.45/0.48	0.70 (0.50–0.99)	REC	0.041	2
*SMAD9*	rs648206	118/127	195/210	104/80	0.48/0.44	1.45 (1.04–2.02)	REC	0.029	61
	rs576434	141/115	191/213	85/89	0.43/0.47	0.74 (0.55–0.99)	DOM	0.046	61
***TGFB1***	**rs8179181**	**269/236**	**119/158**	**24/21**	**0.20/0.24**	**0.72 (0.54–0.96)**	**DOM**	**0.025**	**100**

Note: Significant SNPs after internal validation by bootstrapping analysis (significant in ≥80 runs among 100 runs) are in boldface. WW, homozygous wild-type genotype; WV, heterozygous variant genotype; VV, homozygous variant genotype.

Abbreviations: DOM, dominant model (WW vs. [WV and VV]); REC, recessive model ([WW and WV] vs. VV); ADD, additive model (*P* for trend with increasing number of variant [V] alleles); MAF, minor allele frequency.

*Adjusted by age and ethnicity by unconditional logistic regression.

**The model with the most significant *P* value was defined as the best model for each SNP.

We further explored the cumulative effects of these 13 significant genetic variants in the TGF-β pathway on ovarian cancer risk. Compared with those who carried fewer than 4 unfavorable genotypes, subjects carrying 5–7, 8–10, and 11–13 unfavorable genotypes showed a significantly increased risk with ORs of 2.45 (95%CI, 1.12–5.33; *P* = 0.024), 4.42 (95%CI, 2.04–9.57; *P* = 0.00017), and 6.75 (95%CI, 2.83–16.12; *P* = 0.68×10^−5^), respectively (*P* for trend = 1.67×10^−8^; **Table 2**).

**Table 2:** Cumulative analysis of significant SNPs in TGF-β pathway on ovarian cancer risk.

No. of unfavorable genotypes	Case/control (number of case/number of control)	OR (95% CI)*	*P*	Bootstrapped95% CI
2–4	31/9	1 (Ref.)		
5–7	174/122	2.45 (1.12–5.33)	0.02424	1.13–6.20
8–10	177/220	4.42 (2.04–9.57)	0.00017	2.07–11.18
11–13	32/61	6.75 (2.83–16.12)	1.68×10^−5^	2.78–18.47
*P* for trend			1.67×10^−8^	

Note: Unfavorable genotypes: *BMP2*: rs235757 GG; *INHBC*: rs2228225 AG+GG and rs4760259 (CT+TT); *SMAD1*: rs11724777 TT; *SMAD2*: rs1792689 CT+TT; *SMAD3*: rs10152307 CT+TT and rs7183244 CT+TT; *SMAD6*: rs4147407 CT+TT, rs4075546 AG+GG, rs16953584 GG, and rs4776318 AC+CC; *SMAD7*: rs17186485 AG+GG; *TGFB1*: rs8179181 CT+TT.

*Adjusted by age and ethnicity.

### Gene based analysis for ovarian cancer risk

Gene-based analysis identified *SMAD6* and *TGFB1* (*P*<0.05 for all SNPs examined in each gene using the dominant or additive model; **Table 3**) as genes associated with ovarian cancer risk. *SMAD6* showed the most significant association (*P* = .034), suggesting that of the genes examined genetic variations in this gene had the strongest influence on ovarian cancer risk..

**Table 3:** Global gene *P* values for significant associations between common genetic variations in TGF-β signaling pathway and ovarian cancer risk.

Gene name	Number of SNPs genotyped per gene	*P* for SNPs in gene, by model	
		Dominant model	Additive model
*SMAD6*	29	**.034**	.064
*TGFB1*	6	**.045**	.190
*SMAD7*	21	.074	.147
*INHBC*	5	.125	.137
*AMHR2*	1	.188	.130
*INHA*	4	.208	.640
*ACVR2A*	3	.220	.184
*SMAD2*	8	.223	.264
*SMAD3*	49	.252	.779
*SMAD1*	8	.262	**.050**
*ACVR2B*	3	.374	.465
*SMAD9*	17	.397	.307
*BMP2*	14	.415	.528
*ACVR1B*	3	.481	.743
*BMP4*	5	.570	.724
*NODAL*	6	.580	.558
*SMAD5*	5	.644	.621
*SMAD4*	4	.810	.894
*GDF1*	3	.893	.962
*BMP1*	17	.928	.948
*ACVR1C*	3	.933	.878

Note: Significant *P* values in boldface.

### Haplotype analysis of SMAD6 SNPs

**Figure 1:**
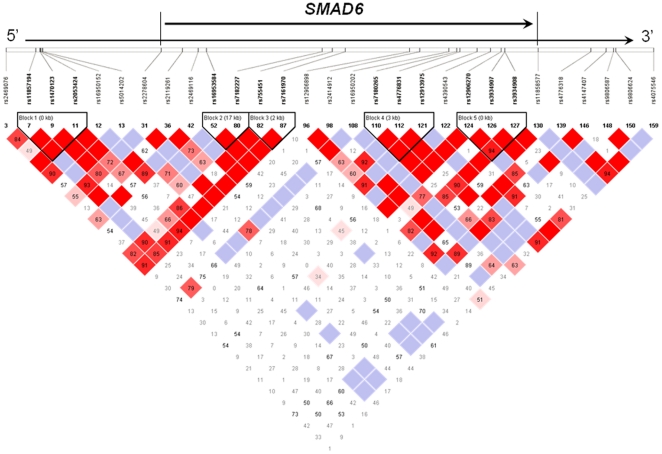
The linkage disequilibrium map of genotyped *SMAD6* SNPs.

As multiple SNPs in the *SMAD6* gene showed significant associations, we performed haplotype analysis for the 29 SNPs genotyped in *SMAD6*. Five haplotype blocks were defined by local linkage disequilibrium (LD) according to HaploView [22] (**Figure 1**, **Table 4**). The definition of “blocks” was described previously by Gabriel et al. [23] We observed significant associations between *SMAD6* haplotypes and risk for ovarian cancer in two LD blocks, block 1 in the 5′ flanking region and block 2 in intron 5 region (**Figure 1** and **Table 4**). Haplotype H2 of block 1 was composed of SNPs rs11857194-rs1470123-rs2053424, and subjects carrying only one variant allele of rs1470123 showed a significant decrease in association with ovarian cancer risk (OR, 0.72; 95%CI, 0.55–0.95; *P* = .018) compared with those carrying the most common haplotype of only one variant allele of rs205342. Haplotype H1 of block 2 comprised of SNPs rs16953584-rs7182227, and subjects carrying only one variant allele of rs16953584 showed a significant 36% reduction in risk (OR, 0.64; 95%CI, 0.44–0.92; *P* = .016) compared with the most common haplotype of two wildtype alleles (**Table 4**)

**Table 4:** Association between *SMAD6* haplotypes and ovarian cancer risk.

*SMAD6*	Control, n (%)	Case, n (%)	Adjusted OR (95% CI)*	*P*
Block1				
H0:W_W_V	318 (48.04)	344 (51.96)	1 (Ref.)	
H1:W_W_W	119 (50.00)	119 (50.00)	0.85 (0.63–1.16)	.303
**H2:W_V_W**	**192 (55.33)**	**155 (44.67)**	**0.72 (0.55–0.95)**	**.018**
H3:V_V_W	205 (48.69)	216 (51.31)	0.95 (0.74–1.21)	.654
Block2				
H0:W_W	622 (48.56)	659 (51.44)	1 (Ref.)	
**H1:V_W**	**79 (58.96)**	**55 (41.04)**	**0.64 (0.44–0.92)**	**.016**
H2:V_V	133 (52.78)	119 (47.22)	0.85 (0.65–1.12)	.247
Block3				
H0:V_W	356 (48.44)	379 (51.56)	1 (Ref.)	
H1:W_W	211 (53.96)	180 (46.04)	0.80 (0.62–1.03)	.081
H2:W_V	265 (49.07)	275 (50.93)	0.99 (0.79–1.24)	.942
Block4				
H0:W_W_W	430 (50.18)	427 (49.82)	1 (Ref.)	
H1:W_V_W	116 (45.85)	137 (54.15)	1.19 (0.89–1.57)	.236
H2:V_V_W	74 (49.01)	77 (50.99)	1.01 (0.72–1.43)	.934
H3:V_V_V	206 (52.02)	190 (47.98)	0.96 (0.75–1.22)	.741
Block5				
H0:W_W_W	308 (49.12)	319 (50.88)	1 (Ref.)	
H1:W_W_V	120 (54.55)	100 (45.45)	0.77 (0.56–1.07)	.117
H2:W_V_W	120 (47.62)	132 (52.38)	1.00 (0.75–1.35)	.976
H3:V_W_V	282 (50.54)	276 (49.46)	0.94 (0.74–1.18)	.575

Note: *Block 1*: rs11857194-rs1470123-rs2053424; *Block 2*: rs16953584-rs7182227; *Block 3*: rs755451-rs7161970; *Block 4*: rs7180265–rs4776831–rs12913975; *Block 5*: rs12906270–rs3934907–rs3934908.

*Adjusted by age and ethnicity.

### Higher-order gene-gene interactions

**Figure 2:**
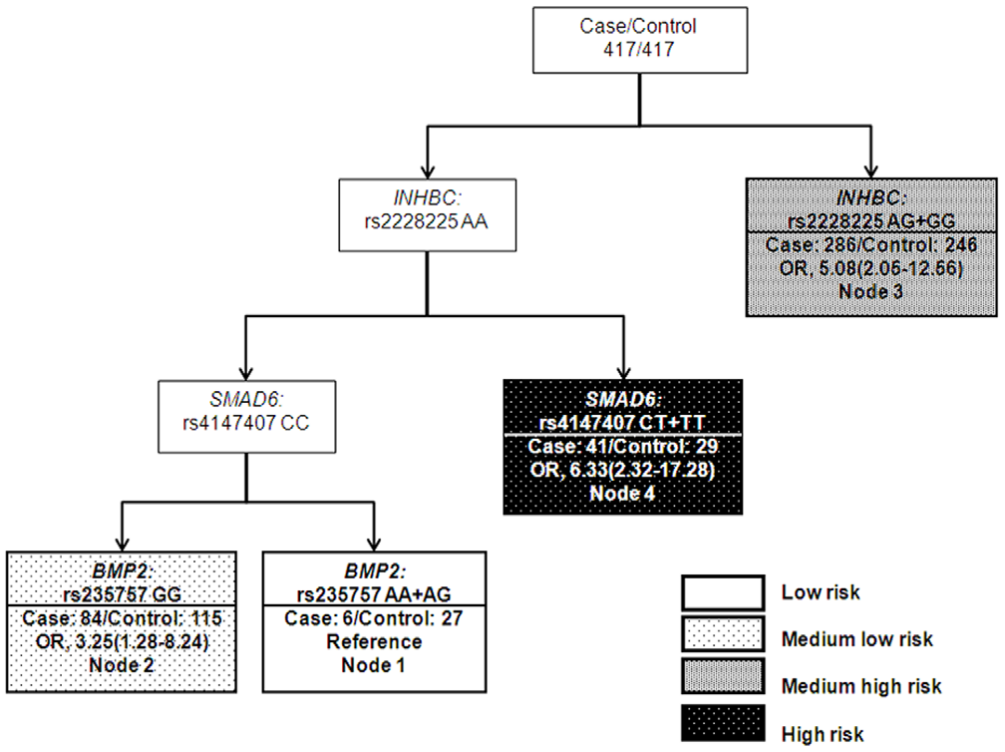
CART analysis of genetic polymorphisms in the TGF-β signaling pathway and risk of ovarian cancer. ORs and 95% CIs (in parenthesis) are presented underneath each terminal node.

CART analysis was applied to explore the higher-order interactions between the 13 significant SNPs. As shown in Figure 2, the tree model resulted in four terminal nodes with different risks for ovarian cancer. The initial split was defined by *INHBC*: rs2228225, indicating that this SNP is the primary factor contributing to variations in ovarian cancer risk in the study population. The reference node of the tree structure was composed of *INHBC*: rs2228225 AA, *SMAD6*: rs4147407 CC, and *BMP2*: rs235757 AA+AG genotypes. Subjects in this node showed the lowest risk of ovarian cancer. The high node was composed of *INHBC*: rs2228225 AA and *SMAD6*: rs4147407 CT+TT and indicated the highest risk of ovarian cancer, with an OR of 6.33 (95%CI, 2.32–17.28; *P* = .0003), suggesting that the genetic variant of *SMAD6*: rs4147407 was a major determinant to switch the trend of lowest risk to highest risk of ovarian cancer (**Figure 2** and **Table 5**).

**Table 5:** Odds ratios of terminal nodes derived from CART analysis for ovarian cancer.

Node group	Control, n (%)	Case, n (%)	Adjusted OR (95% CI)*	*P*	Bootstrap (95% CI)
Low risk (node1)	27 (81.82)	6 (18.18)	1 (Ref.)		
Medium low risk (node 2)	115 (57.79)	84 (42.21)	3.25 (1.28–8.24)	.013	1.38–11.32
Medium high risk (node 3)	246 (46.24)	286 (53.76)	5.08 (2.05–12.56)	4.0×10^−4^	2.21–16.76
High risk (node 4)	29 (41.43)	41 (58.57)	6.33(2.32–17.28)	3.0×10^−4^	2.42–22.14
*P* for trend				1.42×10^−5^	

Note: Node groups are as shown in Figure 2.

* Adjusted by age and ethnicity using unconditional logistic regression.

## Discussion

In this study, we systematically evaluated the associations between a comprehensive panel of genetic variants in the TGF-β pathway genes and ovarian cancer risk. Our results suggested that multiple SNPs in the pathway were associated with ovarian cancer risk. In particular, SNPs in *SMAD6* showed the most significant associations in single SNP and haplotype analyses. Further, there was a cumulative effect of SNPs in the pathway that conferred a significant dose-response trend with subjects carrying the highest number of unfavorable genotypes exhibiting the greatest risk. Results from CART analysis suggested higher-order gene-gene interactions that further defined high vs. low risk subgroups in the study population.

One of the major findings was the significant association of *SMAD6* polymorphisms with ovarian cancer risk. *SMAD6* encodes a protein that is localized in both nuclei and cytoplasm [24] and works as an inhibitory Smad induced by BMPs and TGF-β signals for an auto-inhibitory feedback mechanism in the TGF-β pathway [25], [26]. The *SMAD6* gene is expressed in most human tissues, including the ovary (**Figure S1**). Moreover, *SMAD6* was reported to be overexpressed in ovarian adenocarcinoma compared to normal ovarian tissue [27], and expression of BMP-2 protein has been shown to induce *SMAD6* expression in ovarian cancer cells and was associated with poor prognosis [28]. The function of *SMAD6* in tumorigenesis has not been well established. However, mutations in *SMAD6* have been reported in human ovarian cancer [29]. Since TGF-β signals may function in potent tumor suppression in normal epithelial cells and in early-stage tumors [11], we speculated that genetic variations in *SMAD6* may result in altered gene expression or regulation of signaling function. In this study, four polymorphisms (rs4147407, rs4075546, rs16953584, and rs4776318) in *SMAD6* were found to be significantly associated with ovarian cancer risk. Among these polymorphisms, rs4147407 was associated with increased risk, whereas rs4075546, rs16953584, and rs4776318 were associated with decreased risk. Haplotype analysis further identified two candidate loci of *SMAD6*. Haplotype blocks located in the 5′ flanking region and intron 5 of the *SMAD6* gene respectively, were both associated with decreased risk in this study. However, none of these SNPs are located in the coding region of *SMAD6*, which suggest that these significant SNPs or the identified loci may not directly alter *SMAD6* function but may change the level of gene expression through being located in regulatory regions or being linked to other causal SNPs to affect gene activity. Further *in vitro* and *in vivo* functional studies are needed to characterize the functional significance of the *SMAD6* SNPs identified.

CART analysis revealed gene-gene interactions among *INHBC*, *SMAD6*, and *BMP2*. In the tree model, *INHBC*:rs2228225 was at the initial split, suggesting that this variant functions as the primary risk factor for ovarian cancer. *SMAD6*: rs4147407 was located in the second level of the tree structure and was shown to interact with the *INHBC*: rs2228225 to influence cancer risk. Specifically, the variant alleles of rs4147407 were associated with a 6-fold increase in risk along with common allele of *INHBC*: rs2228225. Indeed, *INHBC* has been identified as beta C chain of inhibin, a hormone that can regulate cell growth and differentiation [30]. The result of CART analysis further strengthened the crucial role of *SMAD6* in influencing the risk of ovarian cancer in the study population.

Our study has some limitations. Chance findings are possible due to small sample size of subgroups. However, we used various statistical methods to control for false positives. For example, we performed bootstrapping analysis for internal validation of the significant SNPs. Other potential limitations include the fact that unmeasured ovarian cancer risk factors in this study (e.g. hormone replacement use) may confound the overall association. Given that we tested a genetic-driven hypothesis rather than an environmental-driven hypothesis, this limitation may be less of a concern. As with all case-control studies, selection bias may also confound the identified associations. Nevertheless, MD Anderson serves as a referral center for many cancer patients from the Kelsey Seybold Clinics in the Houston metropolitan area; therefore our controls are likely to represent the base population that give rise to cancer cases.

In conclusion, our study is the first study to apply a pathway-based approach to evaluate germline genetic variations in the TGF-β pathway and their associations with ovarian cancer risk. We have identified 13 polymorphisms in the TGF-β pathway significantly associated with ovarian cancer risk. In particular, SNPs in *SMAD6* showed the most significant associations. Our data also suggested a cumulative effect of SNPs in the pathway that jointly influenced ovarian cancer risk, and identified higher-order interactions that further define high vs. low risk subgroups in the study population. Future studies are necessary to characterize functional significance of the genetic variants we have identified, as well as to confirm or externally validate the associations in independent populations.

## Supporting Information

Figure S1 Expression of *SMAD6* transcript in human cells and tissues. *SMAD6* tissue expression was referenced in the T1Dbase database, a web-based source for genetic and genomic information on Type I Diabetes (www.t1dbase.org). In this resource, human tissue and cell-type specific gene expression data were obtained from the Novartis GNF SymAtlas database. The expression data were generated on Affymetrix HGU133A chip and a custom microarray.
(DOC)

Table S1 Host Characteristics.
(DOC)

Table S2 Association between tagged SNPs in the TGF-β pathway and ovarian cancer risk.
(DOC)
