# Supplementary material for: Genetic Variants in TGF-β Pathway Are Associated with Ovarian Cancer Risk
Source: PLoS One. 2011 Sep 30;6(9):e25559. doi: 10.1371/journal.pone.0025559 (PMC3184159; doi:10.1371/journal.pone.0025559)
Supplement: Table S1 — Host Characteristics. (DOC) [file pone.0025559.s002.doc]

**Table S1.** Host Characteristics

| Variables | Case, n (%) | Control, n (%) | *P* |
| --- | --- | --- | --- |
| Age, mean (SD) | 60.7 (10.4) | 60.3 (10.7) | 0.554 |
| Total | 417 | 417 |  |
|  |  |  |  |
| Race |  |  |  |
| White | 339 (81.3) | 349 (83.7) | 0.269 |
| Hispanic | 48 (11.5) | 49 (11.8) |  |
| Others | 30 (7.2) | 19 (4.6) |  |
|  |  |  |  |
| Clinical stage* |  |  |  |
| Stage I | 17 (4.6) |  |  |
| Stage II | 23 (6.2) |  |  |
| Stage III | 248 (66.5) |  |  |
| Stage IV | 85 (22.8) |  |  |
|  |  |  |  |
| Histology* |  |  |  |
| Serous | 241 (61.3) |  |  |
| Mixed | 89 (22.7) |  |  |
| Others | 63 (6.0) |  |  |
| * Numbers did not add up to total due to missing clinical  information | | | |
