# Supplementary material for: Genetic Variants in TGF-β Pathway Are Associated with Ovarian Cancer Risk
Source: PLoS One. 2011 Sep 30;6(9):e25559. doi: 10.1371/journal.pone.0025559 (PMC3184159; doi:10.1371/journal.pone.0025559)
Supplement: Table S2 — Association between tagged SNPs in the TGF-β pathway and ovarian cancer risk. (DOC) [file pone.0025559.s003.doc]

**Table S2.** Association between tagged SNPs in the TGF-β pathway and ovarian cancer risk

| Gene | SNP | Position in gene | Allele distribution | Model | OR (95% CI)* | *P* |
| --- | --- | --- | --- | --- | --- | --- |
| ACVR1B | rs2252518 | 3' UTR | C>A | REC | 0.78 (0.49–1.24) | .293 |
|  | rs2854464 | 3' UTR | A>G | REC | 0.80 (0.51–1.26) | .341 |
|  | rs877869 | 3' far gene | C>T | REC | 0.81 (0.50–1.30) | .372 |
| ACVR1C | rs4664229 | 3' UTR | T>C | REC | 0.74 (0.39–1.41) | .356 |
|  | rs7594480 | exon | T>C | DOM | 0.91 (0.62–1.33) | .626 |
|  | rs12993800 | 3' UTR | C>T | DOM | 1.00 (0.75–1.34) | .990 |
| ACVR2A | rs1424954 | 5' near gene | G>A | DOM | 1.25 (0.95–1.65) | .108 |
|  | rs17692648 | 3' UTR | A>C | DOM | 0.88 (0.65–1.18) | .382 |
|  | rs13430086 | 3' UTR | A>T | ADD | 1.02 (0.83–1.24) | .874 |
| ACVR2B | rs7642472 | 3' UTR | C>T | DOM | 1.28 (0.94–1.74) | .112 |
|  | rs3762788 | 5' near gene | A>C | DOM | 1.28 (0.94–1.74) | .113 |
|  | rs12636077 | 3' UTR | G>A | DOM | 1.27 (0.83–1.96) | .273 |
| AMHR2 | rs10876455 | 3' near gene | C>T | DOM | 1.10 (0.82–1.48) | .525 |
|  | rs2002555 | 5' near gene | C>T | DOM | 1.08 (0.80–1.45) | .610 |
| BMP1 | rs4507761 | intron | T>C | DOM | 0.72 (0.48–1.06) | .095 |
|  | rs4872360 | intron | C>T | ADD | 0.87 (0.70–1.08) | .199 |
|  | rs7812993 | intron | A>G | DOM | 0.87 (0.65–1.15) | .312 |
|  | rs6983732 | 3' far gene | C>T | DOM | 0.85 (0.62–1.18) | .338 |
|  | rs4242430 | intron | A>C | DOM | 0.88 (0.67–1.16) | .357 |
|  | rs4075478 | intron | C>T | REC | 1.18 (0.81–1.73) | .382 |
|  | rs2070687 | 5' near gene | C>G | REC | 1.25 (0.69–2.26) | .466 |
|  | rs1124 | 5' near gene | A>G | DOM | 0.91 (0.69–1.20) | .501 |
|  | rs11777932 | intron | A>C | DOM | 0.89 (0.61-–1.30) | .549 |
|  | rs4076873 | intron | A>C | DOM | 0.94 (0.71–1.23) | .635 |
|  | rs3857979 | intron | C>T | REC | 0.94 (0.69–1.30) | .723 |
|  | rs4715 | 5' near gene | A>C | DOM | 0.96 (0.73–1.26) | .774 |
|  | rs11775186 | intron | G>T | DOM | 0.96 (0.66–1.38) | .808 |
|  | rs7819541 | intron | G>T | DOM | 1.04 (0.70–1.56) | .833 |
|  | rs10096722 | 3' far gene | C>G | DOM | 1.04 (0.71–1.52) | .834 |
|  | rs7838961 | intron | A>G | REC | 1.02 (0.75–1.41) | .884 |
|  | rs11996036 | exon | G>A | DOM | 0.98 (0.61–1.57) | .925 |
| BMP2 | rs235757 | 3' far gene | A>G | REC | 0.64 (0.42–0.95) | .029 |
|  | rs6077060 | 5' far gene | C>T | DOM | 0.73 (0.52–1.02) | .065 |
|  | rs235768 | exon | A>T | REC | 0.69 (0.45–1.04) | .076 |
|  | rs6107869 | 5' near gene | A>G | DOM | 1.47 (0.91–2.37) | .117 |
|  | rs173107 | 3' far gene | G>T | REC | 0.74 (0.50–1.10) | .133 |
|  | rs6117432 | 3' far gene | C>T | ADD | 1.18 (0.94–1.48) | .146 |
|  | rs235756 | 3' far gene | T>C | REC | 1.33 (0.90–1.97) | .158 |
|  | rs3178250 | 3' UTR | T>C | REC | 1.52 (0.76–3.04) | .235 |
|  | rs1980499 | 5' far gene | C>T | REC | 0.82 (0.59–1.14) | .242 |
|  | rs1005464 | Intron | T>C | REC | 1.39 (0.74–2.59) | .303 |
|  | rs235710 | 5' far gene | G>A | REC | 1.18 (0.83–1.67) | .358 |
|  | rs235753 | 3' far gene | C>T | DOM | 1.12 (0.85–1.47) | .440 |
|  | rs235751 | 3' far gene | G>A | DOM | 1.10 (0.79–1.53) | .579 |
|  | rs235752 | 3' far gene | T>C | DOM | 1.10 (0.78–1.56) | .582 |
| BMP4 | rs8014071 | 3' far gene | A>G | DOM | 1.27 (0.96–1.69) | .096 |
|  | rs8014363 | 3' far gene | T>C | DOM | 0.83 (0.62–1.11) | .208 |
|  | rs762642 | intron | T>G | DOM | 1.17 (0.89–1.56) | .265 |
|  | rs17563 | exon | C>T | ADD | 1.10 (0.90–1.33) | .365 |
|  | rs10873077 | 3' far gene | C>T | DOM | 1.11 (0.79–1.55) | .558 |
| GDF1 | rs7250622 | intron | A>G | DOM | 1.11 (0.82–1.51) | .485 |
|  | rs757114 | 5' far gene | A>G | DOM | 1.07 (0.75–1.51) | .713 |
|  | rs2075762 | intron | C>T | DOM | 0.95 (0.69–1.30) | .731 |
| INHA | rs7588807 | intron | G>T | DOM | 0.70 (0.50–0.98) | .035 |
|  | rs2059693 | 3' far gene | C>T | REC | 1.36 (0.84–2.18) | .209 |
|  | rs907141 | intron | C>G | DOM | 1.11 (0.84–1.46) | .479 |
|  | rs3731920 | intron | C>T | DOM | 0.95 (0.68–1.34) | .774 |
| INHBC | rs2228225 | exon | A>G | DOM | 1.48 (1.11–1.99) | .008 |
|  | rs4760259 | intron | C>T | DOM | 1.39 (1.06–1.84) | .019 |
|  | rs2242578 | 5' far gene | C>G | ADD | 1.18 (0.96–1.45) | .119 |
|  | rs543410 | 3' far gene | A>G | ADD | 0.95 (0.78–1.16) | .623 |
|  | rs7964492 | 5' far gene | A>C | DOM | 0.97 (0.73–1.29) | .856 |
| NODAL | rs10823547 | 3' far gene | C>G | REC | 1.47 (0.92–2.34) | .105 |
|  | rs2231947 | intron | A>G | DOM | 1.25 (0.93–1.68) | .134 |
|  | rs10762381 | 3' far gene | C>T | ADD | 0.88 (0.73–1.06) | .189 |
|  | rs14761 | 3' UTR | C>T | DOM | 1.21 (0.81–1.81) | .359 |
|  | rs11596795 | 3' far gene | C>T | DOM | 1.12 (0.75–1.67) | .585 |
|  | rs7909303 | intron | G>T | DOM | 1.07 (0.81–1.40) | .649 |
| SMAD1 | rs11724777 | 5' far gene | A>T | REC | 0.63 (0.43–0.91) | .014 |
|  | rs6537355 | 5' near gene | A>G | DOM | 0.69 (0.49–0.98) | .036 |
|  | rs6537356 | intron | A>G | ADD | 1.14 (0.94–1.38) | .175 |
|  | rs11939979 | 3' far gene | A>C | REC | 0.82 (0.60–1.11) | .199 |
|  | rs2118438 | intron | A>G | REC | 0.72 (0.38–1.35) | .301 |
|  | rs13120931 | 3' far gene | T>G | REC | 0.85 (0.61–1.17) | .319 |
|  | rs1016792 | intron | C>T | DOM | 1.12 (0.84–1.50) | .434 |
|  | rs12505085 | 3' far gene | A>G | REC | 1.18 (0.67–2.08) | .576 |
| SMAD2 | rs1792689 | intron | C>T | DOM | 0.69 (0.50–0.95) | .024 |
|  | rs1792658 | intron | A>C | DOM | 0.75 (0.56–0.99) | .043 |
|  | rs4940086 | intron | C>T | DOM | 1.28 (0.97–1.69) | .079 |
|  | rs2000709 | 3' far gene | A>G | DOM | 1.17 (0.86–1.58) | .312 |
|  | rs1792684 | intron | C>T | REC | 0.85 (0.60–1.20) | .362 |
|  | rs1631576 | intron | C>T | DOM | 1.12 (0.83–1.50) | .469 |
|  | rs17814648 | intron | C>T | DOM | 0.87 (0.49–1.55) | .637 |
|  | rs8085335 | 3' UTR | A>G | DOM | 1.02 (0.71–1.47) | .898 |
| SMAD3 | rs10152307 | intron | C>T | DOM | 0.72 (0.54–0.95) | .019 |
|  | rs4776892 | intron | A>T | DOM | 0.73 (0.55–0.98) | .035 |
|  | rs7183244 | intron | C>T | DOM | 0.74 (0.56–0.98) | .035 |
|  | rs7359174 | intron | A>G | DOM | 0.75 (0.54–1.03) | .076 |
|  | rs12102171 | intron | C>T | REC | 0.54 (0.27–1.07) | .077 |
|  | rs17293443 | intron | C>T | DOM | 1.28 (0.96–1.71) | .093 |
|  | rs3809572 | 5' near gene | A>G | DOM | 1.31 (0.93–1.83) | .124 |
|  | rs17293632 | intron | C>T | DOM | 1.25 (0.94–1.66) | .131 |
|  | rs12907997 | intron | C>T | DOM | 1.26 (0.93–1.70) | .139 |
|  | rs7176870 | intron | A>G | REC | 0.77 (0.54–1.09) | .143 |
|  | rs11634793 | intron | C>T | ADD | 1.15 (0.95–1.41) | .152 |
|  | rs7163381 | intron | A>G | DOM | 0.82 (0.62–1.08) | .158 |
|  | rs750766 | intron | A>G | DOM | 1.24 (0.92–1.67) | .167 |
|  | rs1470002 | intron | C>T | REC | 1.30 (0.90–1.88) | .169 |
|  | rs3825977 | intron | C>T | REC | 1.47 (0.81–2.68) | .205 |
|  | rs893473 | intron | C>T | ADD | 0.86 (0.68–1.09) | .212 |
|  | rs4776890 | intron | G>T | REC | 1.28 (0.86–1.89) | .219 |
|  | rs3743342 | 3' UTR | C>T | REC | 1.38 (0.82–2.34) | .227 |
|  | rs9972423 | intron | A>T | REC | 1.28 (0.85–1.94) | .238 |
|  | rs12914140 | intron | C>T | DOM | 0.77 (0.50–1.20) | .253 |
|  | rs2414937 | intron | C>G | REC | 1.47 (0.75–2.88) | .264 |
|  | rs11071933 | intron | C>G | ADD | 0.89 (0.73–1.09) | .273 |
|  | rs11632964 | intron | C>T | DOM | 1.16 (0.87–1.55) | .322 |
|  | rs12916733 | intron | C>T | REC | 1.33 (0.74–2.37) | .340 |
|  | rs1438386 | intron | C>T | ADD | 0.91 (0.75–1.11) | .354 |
|  | rs16950635 | intron | A>G | DOM | 1.22 (0.79–1.89) | .360 |
|  | rs1065080 | exon | G>A | DOM | 0.85 (0.61–1.20) | .360 |
|  | rs4776342 | intron | A>G | DOM | 1.14 (0.86–1.50) | .367 |
|  | rs12900401 | 3' UTR | C>T | DOM | 1.25 (0.77–2.05) | .369 |
|  | rs12324036 | intron | C>T | REC | 0.85 (0.60–1.21) | .369 |
|  | rs1470003 | intron | C>G | REC | 1.16 (0.83–1.62) | .388 |
|  | rs11639295 | intron | C>T | REC | 1.20 (0.78–1.84) | .396 |
|  | rs12904944 | intron | A>G | DOM | 1.11 (0.84–1.46) | .460 |
|  | rs6494633 | intron | C>T | REC | 0.87 (0.60–1.26) | .464 |
|  | rs11637659 | intron | A>G | DOM | 0.90 (0.67–1.20) | .466 |
|  | rs3784681 | intron | C>G | REC | 1.17 (0.75–1.80) | .490 |
|  | rs3743343 | 3' UTR | C>T | DOM | 0.91 (0.69–1.20) | .505 |
|  | rs12915039 | intron | A>C | DOM | 0.92 (0.70–1.21) | .559 |
|  | rs11629568 | intron | G>T | DOM | 0.92 (0.69–1.23) | .587 |
|  | rs7173811 | intron | C>T | DOM | 1.08 (0.80–1.46) | .627 |
|  | rs12913547 | intron | C>T | DOM | 1.07 (0.81–1.41) | .628 |
|  | rs16950553 | intron | A>G | DOM | 1.08 (0.78–1.49) | .640 |
|  | rs12901071 | intron | A>G | REC | 1.09 (0.69–1.71) | .720 |
|  | rs11071939 | intron | C>T | DOM | 0.96 (0.64–1.43) | .836 |
|  | rs17293408 | intron | A>G | DOM | 1.04 (0.71–1.53) | .840 |
|  | rs11071938 | intron | C>T | DOM | 0.98 (0.74–1.29) | .881 |
|  | rs718663 | intron | A>G | DOM | 1.03 (0.70–1.50) | .886 |
|  | rs920293 | intron | C>T | DOM | 1.00 (0.67–1.50) | .987 |
|  | rs17213990 | intron | A>G | DOM | 1.00 (0.71–1.40) | .995 |
| SMAD4 | rs7244227 | 5' far gene | A>G | REC | 1.24 (0.83–1.85) | .292 |
|  | rs948588 | intron | G>A | DOM | 0.83 (0.54–1.26) | .386 |
|  | rs12456284 | 3' UTR | A>G | REC | 1.08 (0.60–1.92) | .805 |
|  | rs1787111 | 5' far gene | T>A | DOM | 0.98 (0.63–1.51) | .912 |
| SMAD5 | rs12719482 | 3' UTR | A>G | DOM | 0.84 (0.62–1.12) | .234 |
|  | rs746993 | intron | A>C | ADD | 0.91 (0.73–1.12) | .367 |
|  | rs12719481 | 3' UTR | A>G | DOM | 0.89 (0.67–1.17) | .390 |
|  | rs3206634 | 3' UTR | C>T | DOM | 0.89 (0.67–1.17) | .390 |
|  | rs3206635 | 3' UTR | A>G | DOM | 0.89 (0.68–1.17) | .409 |
|  | rs17749249 | intron | A>T | DOM | 1.14 (0.84–1.54) | .413 |
|  | rs6886699 | 3' UTR | C>T | DOM | 0.90 (0.68–1.18) | .439 |
|  | rs3764942 | intron | A>G | DOM | 1.05 (0.73–1.51) | .799 |
| SMAD6 | rs4147407 | 3' far gene | C>T | DOM | 1.60 (1.14–2.24) | .007 |
|  | rs4075546 | 3' far gene | A>G | ADD | 0.77 (0.63–0.94) | .010 |
|  | rs16953584 | intron | A>G | REC | 0.45 (0.24–0.87) | .016 |
|  | rs2053424 | 5' far gene | A>C | DOM | 1.43 (1.07–1.92) | .016 |
|  | rs5014202 | 5' far gene | C>T | ADD | 0.78 (0.61–0.99) | .040 |
|  | rs4776318 | 3' near gene | A>C | ADD | 0.82 (0.68–1.00) | .047 |
|  | rs2414912 | intron | C>T | REC | 1.88 (0.96–3.66) | .064 |
|  | rs4390543 | intron | A>G | DOM | 1.33 (0.98–1.81) | .064 |
|  | rs1470123 | 5' far gene | C>G | REC | 0.74 (0.53–1.04) | .079 |
|  | rs2119261 | intron | C>T | REC | 0.74 (0.51–1.07) | .113 |
|  | rs2469116 | intron | C>T | DOM | 1.25 (0.93–1.67) | .140 |
|  | rs3934908 | intron | C>T | DOM | 0.81 (0.60–1.09) | .170 |
|  | rs7161970 | intron | C>T | DOM | 1.20 (0.91–1.58) | .195 |
|  | rs7180265 | intron | A>G | DOM | 0.86 (0.66–1.14) | .300 |
|  | rs755451 | intron | G>T | DOM | 1.17 (0.87–1.57) | .307 |
|  | rs16950152 | 5' far gene | A>T | DOM | 0.84 (0.60–1.18) | .326 |
|  | rs9806624 | 3' far gene | G>T | DOM | 1.19 (0.82–1.73) | .351 |
|  | rs12913975 | intron | A>G | DOM | 0.88 (0.66–1.16) | .364 |
|  | rs16950202 | intron | C>T | DOM | 1.24 (0.77–2.00) | .381 |
|  | rs2278604 | intron | A>C | REC | 1.26 (0.73–2.18) | .407 |
|  | rs9806587 | 3' far gene | C>T | DOM | 1.18 (0.77–1.81) | .454 |
|  | rs11857194 | 5' far gene | C>T | DOM | 1.10 (0.83–1.44) | .513 |
|  | rs2469076 | 5' far gene | T>C | DOM | 1.09 (0.83–1.43) | .547 |
|  | rs4776831 | intron | T>C | REC | 1.09 (0.79–1.51) | .584 |
|  | rs12906270 | intron | A>G | REC | 1.12 (0.72–1.74) | .623 |
|  | rs3934907 | intron | A>C | DOM | 1.08 (0.79–1.46) | .641 |
|  | rs7182227 | intron | C>T | DOM | 0.95 (0.70–1.29) | .755 |
|  | rs11858577 | intron | C>T | DOM | 0.95 (0.65–1.38) | .782 |
|  | rs12906898 | intron | A>G | DOM | 0.97 (0.74–1.28) | .841 |
| SMAD7 | rs17186485 | intron | A>G | DOM | 0.63 (0.42–0.93) | .021 |
|  | rs3736242 | intron | A>G | DOM | 1.37 (1.03–1.81) | .029 |
|  | rs7238442 | intron | C>T | REC | 0.70 (0.50–0.99) | .041 |
|  | rs4939832 | intron | A>G | ADD | 0.81 (0.65–1.01) | .067 |
|  | rs2337143 | 3' far gene | A>G | DOM | 1.29 (0.98–1.70) | .072 |
|  | rs1873190 | intron | C>T | ADD | 0.85 (0.70–1.03) | .092 |
|  | rs6507876 | intron | G>T | DOM | 1.34 (0.94–1.89) | .101 |
|  | rs7351039 | intron | A>G | DOM | 0.71 (0.47–1.08) | .110 |
|  | rs3764482 | intron | C>T | DOM | 0.79 (0.58–1.06) | .112 |
|  | rs6507877 | intron | A>G | REC | 0.75 (0.52–1.09) | .128 |
|  | rs4464148 | intron | C>T | ADD | 0.85 (0.68–1.05) | .130 |
|  | rs16950113 | 3' UTR | C>T | DOM | 0.70 (0.43–1.15) | .159 |
|  | rs4939827 | intron | C>T | DOM | 1.24 (0.91–1.68) | .172 |
|  | rs7227023 | intron | A>G | DOM | 1.27 (0.86–1.86) | .226 |
|  | rs884013 | intron | C>T | DOM | 1.30 (0.83–2.02) | .248 |
|  | rs9945724 | 5' far gene | C>T | DOM | 0.79 (0.53–1.19) | .257 |
|  | rs2337146 | 3' far gene | C>T | DOM | 0.77 (0.45–1.32) | .335 |
|  | rs12456328 | intron | C>T | DOM | 0.86 (0.63–1.18) | .347 |
|  | rs4939830 | intron | A>G | DOM | 0.86 (0.60–1.23) | .411 |
|  | rs12953717 | intron | C>T | DOM | 1.05 (0.79–1.40) | .736 |
|  | rs1316447 | intron | C>T | DOM | 0.96 (0.72–1.28) | .760 |
| SMAD9 | rs648206 | 3' far gene | G>T | REC | 1.45 (1.04–2.02) | .029 |
|  | rs576434 | intron | A>G | DOM | 0.74 (0.55–0.99) | .046 |
|  | rs609418 | 5' far gene | G>T | DOM | 1.31 (1.00–1.73) | .053 |
|  | rs7333607 | intron | A>G | ADD | 0.84 (0.67–1.07) | .154 |
|  | rs12855930 | intron | C>T | REC | 1.30 (0.84–2.02) | .246 |
|  | rs3748305 | intron | A>C | DOM | 0.85 (0.65–1.12) | .255 |
|  | rs9531986 | intron | A>G | DOM | 0.86 (0.65–1.15) | .307 |
|  | rs485033 | intron | A>G | DOM | 1.15 (0.87–1.51) | .338 |
|  | rs17804636 | 3' near gene | A>G | DOM | 1.11 (0.73–1.69) | .624 |
|  | rs1199881 | intron | A>G | DOM | 1.07 (0.76–1.52) | .687 |
|  | rs9576129 | intron | A>G | DOM | 0.95 (0.72–1.27) | .749 |
|  | rs1536654 | intron | A>G | DOM | 1.04 (0.79–1.38) | .780 |
|  | rs7998663 | intron | C>T | DOM | 1.05 (0.72–1.52) | .811 |
|  | rs12877189 | intron | A>G | DOM | 0.96 (0.67–1.39) | .837 |
|  | rs9531987 | intron | C>T | ADD | 0.98 (0.79–1.21) | .846 |
|  | rs511674 | 3' UTR | A>G | DOM | 1.01 (0.54–1.89) | .983 |
|  | rs678037 | intron | A>G | DOM | 0.99 (0.48–2.04) | .986 |
| TGFB1 | rs8179181 | 3' far gene | C>T | DOM | 0.72 (0.54–0.96) | .025 |
|  | rs8110090 | intron | A>G | DOM | 1.42 (0.92–2.19) | .111 |
|  | rs10417924 | 5' far gene | C>T | DOM | 1.26 (0.95–1.68) | .114 |
|  | rs1800472 | exon | C>T | DOM | 1.60 (0.88–2.90) | .124 |
|  | rs4803455 | intron | A>C | DOM | 1.17 (0.86–1.58) | .322 |
|  | rs2241715 | intron | G>T | REC | 0.84 (0.55–1.28) | .414 |

Abbreviations: DOM, dominant model (homozygous wild-type genotype vs. [heterozygous variant genotype and homozygous variant genotype]); REC, recessive model ([homozygous wild-type genotype and heterozygous variant genotype] vs. homozygous variant genotype); ADD, additive model (P for the trend with increasing variant alleles). UTR, untranslated region.

*Adjusted by age and ethnicity
